# Cytokinins résumé: their signaling and role in programmed cell death in plants

**DOI:** 10.1007/s00299-013-1436-z

**Published:** 2013-04-12

**Authors:** A. Kunikowska, A. Byczkowska, M. Doniak, A. Kaźmierczak

**Affiliations:** Department of Cytophysiology, Faculty of Biology and Environmental Protection, University of Łódź, Pomorska 141/133, 90236 Łódź, Poland

**Keywords:** Cytokinin, Cytokinin signaling, Kinetin, PCD

## Abstract

Cytokinins (CKs) are a large group of plant hormones which play a crucial role in many physiological processes in plants. One of the interesting functions of CKs is the control of programmed cell death (PCD). It seems that all CKs-dependent phenomena including PCD are accompanied by special multi-step phosphorelay signaling pathway. This pathway consists of three elements: histidine kinase receptors (HKs), histidine phosphotransfer proteins (HPs) and response regulators (RRs). This review shows the résumé of the latest knowledge about CKs signaling pathways in many physiological processes in plants with special attention paid to PCD process.

## Introduction

Programmed cell death (PCD) is a process that normally occurs during seed germination, development and senescence. This process is crucial for proper functioning of all multicellular organisms, both plants and animals (Kunikowska et al. 2012; Carimi et al. 2003; Collazo et al. 2006). The latest knowledge classifies PCD process on the basis of changes in cell morphology (van Doorn et al. 2011). In animals, apoptosis, autophagy and necrosis are distinguished. In plants, categorization of cell death is more complicated but in 2011 van Doorn et al. proposed the application of morphological criteria to recognize plant cell death thus vacuolar, necrotic and mixed or atypical forms of cell death. Another classification system was also proposed to describe plant PCD. It includes two classes of cell death called “autolytic” and “non-autolytic” which describe processes that occur in intact plants but not in cell cultures (van Doorn 2011).

However, both in animals and plants, changes in the nucleus morphology, especially chromatin condensation and degradation, are the major common morphological features (van Doorn 2011).

Programmed cell death is defined as the genetically determined suicide of individual cells in response to pathogens, environmental stress, and during normal development (Gladish et al. 2006; Collazo et al. 2006) it may be also exogenously induced (Carimi et al. 2003, 2004; Kunikowska et al. 2012).

Up till now, it has been discovered that two natural cytokinins (CKs)—kinetin and benzylaminopurine (BAP or benzyladenine, BA)—at high concentrations are able to induce PCD in plants (Kunikowska et al. 2012; Vescovi et al. 2012; Carimi et al. 2003, 2004). Recent studies have shown that BAP is able to induce PCD in plant cultured cells (Carimi et al. 2003, 2004; Vescovi et al. 2012), whereas kinetin induces this process only in living plant tissues (Kunikowska et al. 2012).

Cytokinins are a large group of plant hormones. They appear to be synthesized in QC (a quiescence center) of roots which seems to be crucial for this synthesis. In cells, CKs are present in chloroplasts or as complexes bound with tRNA (van Staden et al. 2008). These chemical compounds occur endogenously at very low concentrations (pmol g^−1^ FW; Doležal et al. 2007). Cytokinins play a pivotal role at many stages of plant growth and development (Sakakibara 2006; Choi and Hwang 2007). They were discovered during the 1950s as factors essential for cell division (Barciszewski et al. 2007; Mazid et al. 2011) in the presence of auxins (Carimi et al. 2003). Since this discovery, endogenous CKs have been shown as active molecules involved in seed germination, leaf senescence, nutrient mobilization, apical dominance, formation and activity of shoot apical meristem (Mazid et al. 2011) and development of vasculature (Choi et al. 2011). They also promote seed germination, starch and chlorophyll production, bud differentiation and branching (Doležal et al. 2007). Cytokinins influence plant shape depending on environmental factors, such as light, water and nutrition (Hirose et al. 2008). There is also evidence that CKs are involved in the modulation of metabolism and morphogenesis during environmental stress (Hirose et al. 2008).

It seems that CK receptors are involved in many physiological processes including CK-dependent PCD. These receptors could act as a multi-step phosphorelay system which functions like histidine kinase (HK; Schaller et al. 2011).

## Characteristic of natural and synthetic cytokinin properties

Cytokinins are N^6^-substituted adenine derivates. Isopentenyl pyrophosphate is the starting compound in CK synthesis pathway (Sakakibara 2006). Depending on N^6^-substituent, CKs are classified as isoprenoid (e.g. zeatin) or aromatic compounds (e.g. BAP; Doležal et al. 2007). In plant cells, CKs are usually bound with β-d-ribose or β-d-glucose forming ribosides. Free adenine backbones are biologically the most active forms of CKs (Griffaut et al. 2004). However, biological activity of CKs depends mainly on the N^6^-substituent (Spíchal et al. 2004).

There are natural and synthetic CKs, but this classification is not clear. For instance, kinetin and BAP are sometimes called synthetic CKs, because in plants their amounts are so small that for commercial purposes they must be synthesized in chemical processes (van Staden et al. 2008). However, both kinetin and BAP were discovered in plants (Barciszewski et al. 2007), so in this paper we classified them as natural compounds.

Natural cytokinins may occur in different forms which include free bases, glucosidic conjugates, ribosides or nucleotides (van Staden et al. 2008). Among naturally occurring CKs, there are compounds with an aliphatic substituent and this group of CKs is represented by isopentenyladenine (iP), zeatin (*trans*- and *cis*- isomers) and their derivates. Derivates of zeatin and iP as well as their sugar conjugates are most popular, but their occurrence depends on plant species, stage of development and tissue (Sakakibara 2006). Accordingly, *trans*-zeatin and iP are main forms in *A. thaliana*, while *cis*-zeatin predominates in rice, chickpea and maize (Sakakibara 2006).

Zeatin, an isoprenoid CK, and its derivatives are the best described CK group (Gajdošová et al. 2011). Zeatin also occurs in nature as glucosidic conjugates as well as metabolites, e.g. dihydrozeatin which also exhibits CK activity (van Staden et al. 2008).

BAP, kinetin and *o*- and *m*-topoline are CKs which have an aromatic substituent (Barciszewski et al. 2007; Kudo et al. 2012).

Benzylaminopurine and its derivates are active and readily available substances which stimulate growth and metabolism in plants; they are also commonly used in plant biotechnology (Doležal et al. 2007) and in micropropagation (van Staden et al. 2008). In different cultivars of bananas, BAP has recently been shown to induce shoot tip multiplication and to stimulate growth of axillary and adventitious buds and foliar development of shoot tip cultures (Jafari et al. 2011).

Kinetin (6-furfuryladenine), a purine-derived CK, discovered as a degradation product of DNA, plays a crucial role in plant cell division. It was isolated by Professor Scoog in 1955 and now it seems that it is the best-known CK (Barciszewski et al. 2007; van Staden et al. 2008). Kinetin was recognized as a synthetic by-product (Barciszewski et al. 2007) of herring sperm DNA autoclaving which suggested that it was not a natural compound (Minorsky 2003). However, in 1996, it was discovered that this CK occurred in commercially available DNA from human cells, human urine and from plants (Barciszewski et al. 2007). Natural kinetin was also identified in *Casuarina equisetifolia* root nodules nodulated by *Frankia* as well as in liquid endosperm of fresh young coconut fruits (Barciszewski et al. 2007).

Most experimental data showed strong antioxidant properties of kinetin and it was found to protect DNA against oxidative damage in Fenton reaction (Minorsky 2003). Nowadays, it is used in the rosacea therapy and plays a pivotal role in cosmetology because of its anti-aging properties (Wu et al. 2007).

Synthetic CKs and some synthetic compounds have biological activity similar to natural CKs, some of them also have similar chemical construction, but the others exhibit only CK activity (Yonova 2010). Amino purine and non-amino purine synthetic CKs are known (van Staden et al. 2008).

Researchers created some synthetic compounds exhibiting CK activity (van Staden et al. 2008) because natural CKs are not used in commercial laboratories due to their cost. Mostly, they are N^6^-substituted adenine derivates, but they can also be less chemically related compounds, like 4-alkylaminopteridines or 6-benzyloxypurines (van Staden et al. 2008). Non-purine CKs are the biggest group of synthetic compounds with CK activity (Yonova 2010). This group contains the following subgroups: benzimidazoles, pyrimidines, *O*-6-substituted derivates of hypoxanthine, guanine and the most significant subgroup—aromatic ureas.

## The way of perception and transduction of CK signals *in planta*

Cytokinin receptors belong to a class of HK receptors (Shi and Rashotte 2012). These receptors act similarly to a two-component signal transduction system which resembles the typical model of a two-component system widely used by bacteria (Beier and Gross 2006) and some fungi (Schaller et al. 2011).

**Fig. 1:**
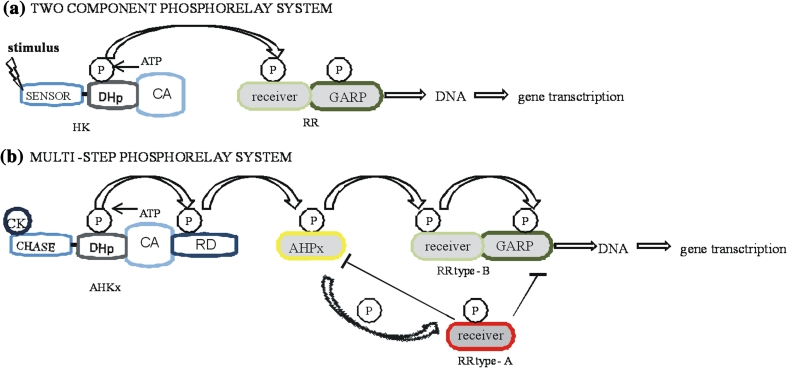
**a** Two-component signaling system consists of a histidine kinase receptor (HK) and a response regulator (RR). The kinase receptor autophosphorylates a conserved histidine residue in response to a stimulus perceived by a sensor domain of HK. A phosphoryl group is transferred to a conserved aspartate residue of a receiver domain of the RR. The active RR subsequently triggers a response, usually acting as a transcription factor. DHp domain is a phosphodonor and mediates dimerization. CA domain is responsible for ATP binding and catalyzes autophosphorylation. CK-cytokinins, P-phosphoryl group AHKx: AHK2, AHK3, AHK4, DHp-histidine phosphotransferase domain, CA-catalytic domain, RD-receiver domain, AHPx: AHP1, AHP2, AHP3, AHP5; **b** A multi-step phosphorelay signaling system in *Arabidopsis thaliana* consists of a hybrid histidine kinase receptor (AHK), a histidine phosphotransfer protein (AHP) and a response regulator (ARR). Cytokinins are perceived by CHASE domains of AHK receptors localized both in plasma membrane and ER. CA domain is responsible for ATP binding and catalyzes autophosphorylation of a conserved histidine residue. DHp domain is a phosphodonor and mediates dimerization. RD shuttles phosphor to HP. Finally, HP moves phosphoryl group to a conserved aspartate of RR localized in the nucleus. Type-B RRs are transcription factors which switch on the expression of CK-regulated genes (including type-A RRs). Type-A RRs are engaged in repressing the CK signaling

In the prototypical two-component system, the receptors consist of two proteins, a sensor and a response regulator (RR) (Fig. 1). Active forms of receptors are homo- or heterodimeric combinations. The sensor is made up of two domains: external and internal. The external domain is responsible for ligand binding and signal perception (Jeon and Kim 2012), whereas the internal domain, histidine phosphotransfer proteins (HPs), undergoes autophosphorylation and then subsequently transfers phosphoryl groups to RRs i. e. to downstream transcriptional activators or repressors in a nucleus where they can affect changes in cellular physiology (often by regulating gene expression; Laub and Goulian 2007).

A common variant of the canonical two-component signaling pathway was characterized in a number of plant species such as *A. thaliana* (Nishimura et al. 2004), *Zea mays* (Lomin et al. 2011; Spíchal et al. 2004), *Oryza sativa* (Ito and Kurata 2006), *Medicago truncatula* (Gonzalez-Rizzo et al. 2006) and *Glycine max* (Mochida et al. 2010). In plants, the two-component CK-signaling pathway is called multi-step phosphorelay.

Numerous examples indicate that the multi-step phosphorelay CK signaling plays a huge role in many physiological processes in the plant kingdom, including molecular, cellular and developmental regulations since embryo creation to tissue formation (shoot and root apical meristems, stem and root vascular systems, nodule) and organismal response to stress, pathogens, senescence (Hwang et al. 2012).

**Fig. 2:**
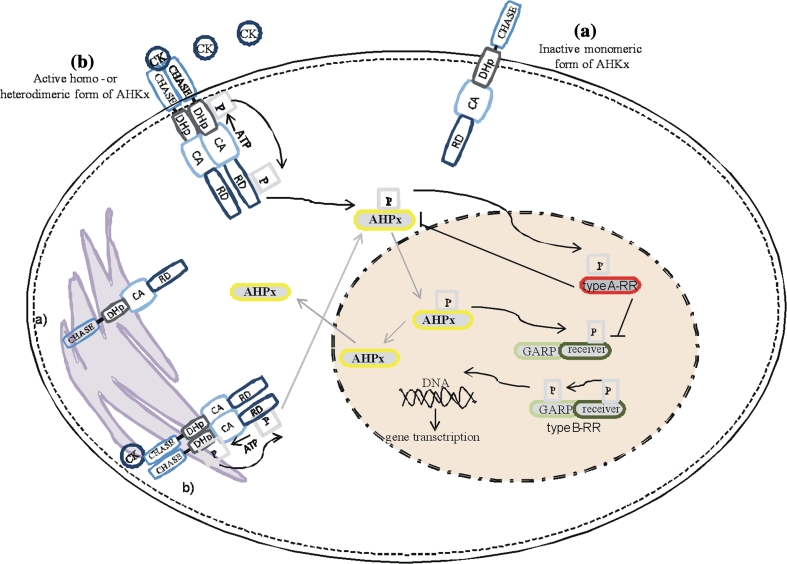
A model of cytokinin multi-step phosphorelay signaling system in *Arabidopsis thaliana*. **a** Inactive form of histidine kinase receptor (AHKx). **b** Active form of histidine kinase receptor after ligand (CK) binding (mono- or heterodimeric combination of AHKs). For details, see Fig. 1 legend

Cytokinin signals are perceived by the extracellular input domain of hybrid HK receptor named CHASE sensing domain (Kieber and Schaller 2010). Histidine kinases are hybrids because they have an additional receiver domain (Table 1). CHASE domain appears in mosses, lycophytes and higher plant. After ligand binding, the internal domain of the receptor, the histidine domain, undergoes autophosphorylation on a conserved histidine residue. Then, the high-energy phosphoryl group is transported to an aspartate histidine residue of the receiver domain of a histidine receptor (Bijlsma and Groisman 2003), after that the phosphoryl group is transferred by HP to an aspartate residue in *N*-terminal of receiver domain of RR (Tables 2, 3; Laub and Goulian 2007). Output domains of the RRs named GARP are activated after phosphorylation of the receiver domain and are often involved in induction of core signaling components (Hwang et al. 2012). For details, see Fig. 2.

**Table 1:** AHK and AHP engaged in CK signaling, their interaction, function and localization

Elements engaged in CK-signaling	Interaction between elements engaged in CK-signaling	Functions of the elements engaged in CK-signaling	Localization *in planta* and plant cells
AHK			
AHK2	ARR2	Increases CK-dependent ARR6 promoter activity, engaged in cold response	Root and leaf vasculatures, shoot meristems, root tips stems, flowers
AHK3	ARR3	Increases CK-dependent ARR6 promoter activity senescence and root development, engaged cold response	Root and leaf vasculatures, shoot meristems, root tips, stems, flowers
AHK4	AHP2/AHP5 > AHP3	Increases CK-dependent ARR6 promoter activity, engaged in senescence and root development, controls root meristem size, PCD	Root and leave vasculatures, shoot meristems, root tips, stems, flowers
AHP			
AHP1	ARR1, ARR2, ARR4, ARR9 and ARR10	Positive regulators of CK signaling, translocates signals from cytoplasm to nucleus upon CK treatment	Leaves, roots, seedlings
AHP2	ARR1, ARR2, RR10, AHK4	Positive regulators of CK signaling, translocates signals from cytoplasm to nucleus upon CK treatment, positive factor in cold response	Roots, leaves, stems, seedlings, flowers
AHP3	ARR1, ARR10, AHK4, ARR9	Positive regulators of CK signaling, positive factor in cold response	Roots, leaves, stems, seedlings, flowers
AHP5	ARR1	Translocates signals from cytoplasm to nucleus upon CK treatment	Roots, leaves, stems, flowers

To et al. (2004); Hwang et al. (2012); Argyros et al. (2008); Heyl and Schmülling 2003; Hirose et al. (2008); Tajima et al. (2004); Mason et al. (2004); Kiba et al. (2003); Kiba et al. 2002; Jeon and Kim (2012); Zalabák et al. (2012); Shi and Rashotte (2012)

**Table 2:** ARR type-A, negative, regulators of cytokinin signaling engaged in CK signaling, their interaction, function and localization (for references see Table 1)

Elements engaged in CK-signaling	Interaction between elements engaged in CK-signaling	Functions of the elements engaged in CK-signaling	Localization *in planta* and plant cells
ARR3	ARR9	Regulation of petiole elongation	Root and leaf vasculatures
ARR4	AHP1	Represses CK induced transcription of ARR6, renders tissues more CK sensitive, induced by osmotic stress, regulates of petiole elongation, engaged in environmental stresses such as drought, salt etc., signaling module in cytokinin and light signal transduction pathways	Root and leaf vasculatures
ARR5	No data	Represses CK induced transcription of ARR6, engaged in osmotic stress and environmental stresses such as drought, salt etc.	Shoot and root meristems, leaves, fruit abscission zones
ARR6	No data	Represses CK induced transcription of ARR6	Roots, leaf and shoot vasculatures, root meristems
ARR7	No data	Represses CK induced transcription of ARR6	Inflorescence, shoot apical meristem,
ARR8	No data	Renders transgenic overexpression CK insensitive, engaged in osmotic stress	Root tips, root meristems, root vasculatures, leaf vasculatures, anthers
ARR9	AHP1, AHP3 and ARR3	No data	Root tips, root meristems, root vasculatures, leaf vasculatures
ARR15	No data	No data	Root tips
ARR16	No data	No data	No data
ARR17	No data	No data	No data
ARR20	No data	No data	Pistils, flowers, leaf vasculatures, shoot meristems

**Table 3:** ARR type-B, positive, regulators of cytokinin signaling engaged in CK signaling, their interaction, function and localization (for references see Table 1)

Elements engaged in CK-signaling	Interaction between elements engaged in CK-signaling	Functions of the elements engaged in CK-signaling	Localization *in planta* and plant cells
ARR1	AHP1, AHP2 and AHP3	Activates transcription of CK response genes, its overexpression causes aberrant cell proliferation essential and redundant roles during cytokinin signaling	Young leaves, anthers, root tips
ARR2	AHK2 and AHK3 AHP2	Activates transcription of CK response genes, its overexpression promotes cell proliferation and shoot growth, engaged in cold response	Pollens, leaf and root vasculatures, young leaves, shoot meristems, root tips
ARR10	AHP1, AHP2, AHP3	Activates transcription of ARR6, the head of a transcriptional cascade to regulate the cytokinin response	Roots, leaf vasculatures, shoot meristems
ARR11	AHP2	Binds DNA specifically and activates transcription overexpression causes aberrant cell proliferation	Shoot meristems, young leaves
ARR12	No data	The head of a transcriptional cascade to regulate the cytokinin response	Young leaves, developing seeds and roots, leaf vasculatures
ARR13	No data	No data	Young leaves and flowers, leaf vasculatures
ARR14	No data	No data	Young leaves
ARR18	No data	No data	Young leaves and flowers, flowers, shoot meristems
ARR19	No data	No data	Trichome
ARR20	No data	No data	Young leaves, leaf vasculatures, shoot meristems, pistils

The multi-step phosphorelay system engaged in CK signaling is well known in *A. thaliana*. There are three *A. thaliana* HKs (AHK2, AHK3, AHK4) which are responsible for reception of the CK signals (Higuchi et al. 2004). Moreover, three *A. thaliana* HPs (AHP1, AHP2 and AHP5) that mediate phosphoryl group transfer between AHKs and ARRs were also characterized (Punwani et al. 2010). These AHPs act as positive regulators of CK signaling (Hutchison et al. 2006). They occur both in cytoplasm and nucleus (Grefen and Harter 2004). The RRs, final elements of CK perception and signal transduction, are divided into 4 subgroups i. e. type-A, type-B, type-C and APRRs (Arabidopsis pseudo-RRs). Type-A and type-B are involved in the CK-signaling pathway (Mizuno 2004).

Type-A ARRs consist of 10 RRs -3, 4, 5, 6, 7, 8, 9, 15, 16, 17. These RRs are small proteins. They carry an Asp-containing receiver domain and a short C-terminal output domain. They function as primary transcriptional targets of CKs; their transcripts are accumulated fast after CKs treatment (Mizuno 2004). Most of type-A ARRs were found to be localized in the nucleus. They were reported to be mainly negative regulators of CK signaling (To et al. 2007).

Type-B ARRs consist of 11 RRs -1, 2, 10, 11, 12, 13, 14,18, 19, 20 (Argueso et al. 2012). They have longer C-terminal receiver domains than type-A ARRs (Argyros et al. 2008), binding with DNA and a trans-activating motif (To et al. 2007). They play a role of positive regulators of CK signaling (Hwang et al. 2012) which monitor the transcription of many CK-regulated targets including the induction of type-A ARRs (To et al. 2007; Rashotte et al. 2006).

The specific CK signaling multi-step phosphorelay system was also characterized in *M. truncatula* (MT, Gonzalez-Rizzo et al. 2006), where one HK (MtCRE1) and two RRs of type-A (MtRR4) and type-B (MtRR1) were identified. This CK-signaling pathway was observed during development of lateral roots and during symbiotic nodulation (Ariel et al. 2012).

The CK-signaling pathway was also described in *O. sativa*, where four HKs (OHK2, OHK3a, OHK3b, OHK4 and OHK5) were observed. In rice, only two of HPs (OHP3 and OHP4) are involved in the CK signaling multi-step phosphorelay system. The RRs present in rice are also divided into two groups: OsRRs and ORRs. The former is similar to type-A ARRs (Doi et al. 2004), which act as negative regulators of CK-signaling pathway. This group contains OsRR4, OsRR8, OsRR9, OsRR10, OsRR12, OsRR13. The latter group includes seven ORR: ORR1, ORR2, ORR3, ORR4, ORR5, ORR16, ORR7. These proteins act as type-B ARRs and control transcription of other factors involved in the CK signal reception (Tsai et al. 2012; Ito and Kurata 2006).

Similarly as in the above-mentioned species, putative forms of CK receptors, CK HP and CK RRs occur in *G. max.* Researchers classified *G. max* CK receptors into eight classes: GmHK11, GmHK12, GmHK13, GmHK14, GmHK15, GmHK16, GmHK17. While the HP described in soybean, i. e. GmHP01–06, GmHP09 and GmHP10 are functionally close to the CK positive regulators AHP1, AHP2, AHP3 and AHP5. The other two HK proteins, GmHP07 and GmHP08, are in close relationship to AHP4, and they might be negative regulators of CK signaling. The RRs engaged in CK signaling appearing in soybean are reported to be type-A ARRs and type-B ARRs. Type-A RRs (GmRR01, GmRR02, GmRR03, GmRR06, GmRR09, GmRR10, GmRR18) were described as negative regulators of CK signaling, whereas type-B (GmRR25, GmRR26, GmRR29) seems also to play a positive role in transcription factors engaged in CK signaling (Mochida et al. 2010).

Cytokinin-responsive His-protein kinases were also identified in *Z. mays* (ZmHK1, ZmHK2, ZmHK3a). They were closely related to AHK4, AHK3, and AHK2 receptors, respectively (Lomin et al. 2011). Three HPs (ZmHP1, ZmHP2, ZmHP3) and ten RRs (ZmRR1 to ZmRR10) were also detected in *Z. mays*. In this plant, HPs also play a significant role in signal integration and their transduction between HK and RR (Yonekura-Sakakibara et al. 2004). In maize, RRs which regulate cellular responses to CKs (Asakura et al. 2003) can be also classified into two types: type-A of RRs (ZmRR1, ZmRR2 and ZmRR4 to ZmRR7) and type-B of RRs (ZmRR8–ZmRR10).

## Cellular and sub-cellular localization of multi-step cytokinin signaling components

Cytokinin receptors have been widely studied (Kieber and Schaller 2010; Dortay et al. 2008; Gupta and Rashotte 2012). Their biochemical properties and specific functions were examined, but their cellular localization is still not fully investigated. Researches focused on AHK3 and AHK4 in *A. thaliana*. Firstly, Kim et al. (2006) reported that a CK signal was perceived by AHK3 at the plasma membrane. However, later it was indicated that AHK3 and AHK4 were mostly localized in ER (Wulfetange et al. 2011) or both in plasma membrane and endoplasmic reticulum (Shi and Rashotte 2012; Nongpiur et al. 2012).

The AHP proteins were found to be localized both in cytoplasm and nucleus. It seems that they undergo bulk re-localization between nucleus and cytoplasm. However, their relocalization seems to be independent of the CK-signaling pathway (Punwani et al. 2010).

Localization of A- and B-type RR proteins was only detected in a nucleus, apart from two type-A ARRs, ARR3 and ARR16, for which localization in the cytosol was described. Nonetheless, a low signal of these two above-mentioned RRs could be also detected in the nucleus (Dortay et al. 2008).

## Role of cytokinin and their signaling during programmed cell death

Programmed cell death process is apparently under hormonal control. Plant hormones such as ethylene, brassinosteroids and CKs together with other signaling compounds regulate PCD process in a complex way (Gadjev et al. 2008). It is well known that in cereal aleurone cells, PCD is induced by gibberellins, while, ABA blocks the effect of gibberellins and delays this process (Carimi et al. 2003). Recently, studies have shown that high levels of CKs such as BAP and kinetin (Carimi et al. 2004; Kunikowska et al. 2012; Vescovi et al. 2012) are also able to induce PCD in plant tissues and cell cultures (Carimi et al. 2003, 2004; Kunikowska et al. 2012). High concentration of BAP (27 μM) induced PCD in cell cultures of carrot (*Daucus carota*) and *A. thaliana* (L.) Heynh. In both carrot and Arabidopsis, PCD was induced by accelerating senescence or senescence-like process both in vitro (in cell cultures) and in vivo (in leaves; Carimi et al. 2004; Vescovi et al. 2012), but the oligo nucleosomal nuclear DNA fragmentation—one of the PCD hallmarks (Palavan-Unsal et al. 2005)—was observed only in cell cultures. This observation suggested that cell cultures might be used as a model system to study senescence (Carimi et al. 2004). Additional PCD hallmarks induced by BAP include chromatin condensation and release of cytochrome c. The DNA fragmentation in carrot was detected at lower concentration of BAP (13 μM; Carimi et al. 2003). The BAP-induced PCD process was accompanied by decreased cell growth and blocked cell division; however, it seems that BAP might induce PCD not only by limiting cell proliferation (Carimi et al. 2003).

More recently, it has been reported that kinetin is able to induce PCD *in planta*, i.e. in root cortex cells of *Vicia faba* ssp. *minor* (Kunikowska et al. 2012), but not in human and animal cells (Berge et al. 2006; Ishii et al. 2002). Double-colored staining with acridine orange (AO) and ethidium bromide (EB) showed that kinetin induced cell death in mid cortex cells, but not in meristem. The activity of dehydrogenases secreted from mitochondria (about 40 %) was correlated with the amount of living root cortex cells (about 40 %) and the number of retained mitochondria (about 45 %). There are also characteristic morphological changes in nuclei which include chromatin condensation, micronuclei formation, invagination, chromatin degradation and fragmentation of nuclei, which was shown both by AO/EB and DAPI staining. Kinetin also decreases root lengths and simultaneously increases their weight and width. Moreover, it induces acidic vacuole formation as well as increases the amount of calcium ions. Production of ROS is also observed (Kunikowska et al. 2012).

Other studies of CKs, e.g. on cultured cells of *A. thaliana* and *D. carota* showed that zeatin did not induce a PCD process (Carimi et al. 2004). It may result from the fact that chemical structures of BAP, kinetin and zeatin are different so these compounds have varied ability to induce PCD (van Staden et al. 2008; Carimi et al. 2004; Kunikowska et al. 2012).

The question arises *how CKs are able to induce this process.* There are CK receptors acting as HK, CK HP and CK RR that create a specific system of reception and transduction of CK signals named multi-step phosphorelay system (Ferreira and Kieber 2005). Some results indicate that one of the *A. thaliana* CK receptors (AHK4) is engaged in CK-stimulated induction of PCD (Vescovi et al. 2012) This discovery confirmed a central role of AHK4 and excluded participation of two other AHKs named AHK2 and AHK3 in mediating CK (BAP)-induced PCD. Moreover, it has been explained why such a high level of CKs is necessary to induced PCD. Vescovi et al. (2012) showed that AHK4 had low affinity to applied CK. It seems that HK receptors may be also engaged in the mechanism of kinetin-induced cell death (Kunikowska et al. 2012). It has been proposed that phosphoribosyl transferase converts kinetin to monophosphates (Kunikowska et al. 2012), purine ligands specific for HKs receptors (AHK2, AHK3 and AHK), which were discovered in plasma membrane and endoplasmic reticulum membranes of *Zea mays* and Arabidopsis (Caesar et al. 2011).

## Future directions

Subcellular AHK localization sheds a new light on hormone functioning (Caesar et al. 2011) during PCD process (Vescovi et al. 2012). The ER localization may explain why CK signal is perceived by different subcellular localizations and through distinct CK metabolites (Wulfetange et al. 2011). Unfortunately, there are still no reports concerning the role of the other two elements (AHPs and ARRs) of the CK-signaling multi-step system in PCD process. Recently, multi-step signal transduction system has been described in a lot of plant species (Vescovi et al. 2012; Lomin et al. 2011). It is involved in many physiological processes, and thus it is possible to create models of the remaining elements of the system engaged in PCD and of their interactions. Some morphological and metabolical features observed during PCD in root cortex cells of *V. faba* ssp. *minor* after kinetin treatment (Kunikowska et al. 2012) might be related to the multi-step signal transduction system.
